# Time- and dose-dependent activation of the NLRP3 and MyD88/NF-κB pathways by *Trueperella pyogenes* membrane vesicles in bovine endometrial epithelial cells

**DOI:** 10.1186/s13567-025-01587-9

**Published:** 2025-07-21

**Authors:** Dengfu Li, Haixia Li, Zhu Wang, Yuchen Huang, Chenchong Zhao, Hongxia Zhang, Yanan Zhao, Naihui Zhu, Xirong Tang, Yaping Jin, Dong Zhou

**Affiliations:** 1 College of Veterinary Medicine, Northwest A&F University, Xianyang, China; 2 Key Laboratory of Animal Biotechnology, Ministry of Agriculture and Rural Affairs, Northwest A&F University, Xianyang, China

**Keywords:** *Trueperella pyogenes*, membrane vesicles, NLRP3 inflammasome, MyD88/NF-κB, endometritis

## Abstract

*Trueperella pyogenes* is an opportunistic pathogen frequently associated with bovine endometritis, yet the mechanisms by which it induces uterine inflammation remain incompletely understood. In this study, we investigated the effects of *T. pyogenes* and its membrane vesicles (MVs) on bovine endometrial epithelial cells (BEECs) and explored the underlying inflammatory pathways involved. Bovine endometrial epithelial cells (BEECs) were treated with *T. pyogenes* (MOI = 100) or MVs at various concentrations (1 × 10^8^, 1 × 10^7^, or 1 × 10^6^ particles/mL) for 6–24 h. Inflammatory cytokines (IL-1β, IL-6, IL-18, or TNF-α) and the activation of the NLRP3 and MyD88/NF-κB signalling pathways were analysed by ELISA, qRT‒PCR, and western blotting. Cell death mechanisms were assessed by flow cytometry and scanning electron microscopy. *T. pyogenes* significantly upregulated inflammatory cytokine mRNA expression at 6 and 12 h and protein expression at 12 and 24 h. Compared with bacterial stimulation at 12 h, MVs induced earlier activation of the NLRP3 inflammasome at 6 h. High-concentration MVs induced necrosis-like membrane disruption, whereas moderate concentrations promoted apoptosis and pyroptosis. Both *T. pyogenes* and its MVs activated the MyD88/NF-κB signalling pathway, with significantly increased phosphorylation of P65 at 12 h. Cytokine secretion exhibited time- and dose-dependent trends, aligning with transcriptional changes. Collectively, these findings demonstrate that *T. pyogenes* MVs contribute to endometrial inflammation through the NLRP3 and MyD88/NF-κB signalling pathways, with distinct forms of cell death determined by MV concentration. These findings highlight MVs as key virulence factors and potential therapeutic targets for bovine endometritis.

## Introduction

Endometritis is characterized by infection or inflammation of the endometrium. It often occurs after calving and affects the reproductive performance of dairy cows [1–3]. Cows with endometritis show delayed ovarian activity, longer calving-to-conception intervals, and reduced conception rates [4, 5]. These issues result in economic losses due to treatment costs, discarded milk, and reduced productivity. In severe cases, affected cows may be culled early [2, 6].

*Trueperella pyogenes* (*T. pyogenes*) is a common pathogen in postpartum uterine infections. It causes purulent inflammation and is frequently isolated from cows with endometritis [7]. During calving, bacteria such as *Escherichia coli*, *Staphylococcus aureus, Streptococcus pyogenes*, and *T. pyogenes* may enter the uterus through the birth canal [8]. *T. pyogenes* expresses several virulence factors. Pyolysin (PLO), encoded by the *plo* gene, can damage host cells. Other factors, such as fimbriae, collagen-binding proteins, and neuraminidases, support bacterial adhesion and colonization [7, 9]. Previous studies have shown that recombinant PLO can trigger inflammatory responses and activate the NLRP3 inflammasome [10].

Bacterial membrane vesicles (MVs) are small, spherical structures released during bacterial growth [11]. MVs range in size from 40 to 400 nm, function as carriers of bioactive molecules, including proteins, nucleic acids, and toxins, and are increasingly recognized as important vehicles for host‒pathogen interactions [12, 13]. Several studies have shown that MVs can trigger strong inflammatory responses. For example, MVs from *Staphylococcus aureus* induce TNF-α release in human keratinocytes via Toll-like receptors (TLRs), whereas *E. coli*-derived MVs activate the NF-κB pathway and coagulation cascades in endothelial cells [14, 15]. *Porphyromonas gingivalis* MVs can penetrate host tissues and disrupt epithelial integrity, amplifying inflammatory signalling [16, 17]. These findings suggest that MVs not only function in bacterial communication but also serve as potent virulence factors capable of modulating host immune responses.

While *T. pyogenes* has been established as a major contributor to uterine inflammation, the role of its membrane vesicles in bovine endometritis remains unexplored. In this study, we investigated the inflammatory response of bovine endometrial epithelial cells (BEECs) to *T. pyogenes*. We then evaluated the effects of different concentrations of *T. pyogenes*-derived MVs on BEECs, with a focus on the activation of the NLRP3 inflammasome and MyD88/NF-κB signalling pathways. Finally, we assessed the modes of cell death induced by *T. pyogenes* and its MVs using flow cytometry and scanning electron microscopy. This work provides novel insights into the pathogenic mechanisms of *T. pyogenes* and identifies bacterial MVs as potential therapeutic targets for the prevention and treatment of bovine endometritis.

## Materials and methods

### Culture of *T. pyogenes* and extraction of MVs

*T. pyogenes* were cultured as previously described [18]. Briefly, *T. pyogenes* was cultured in brain heart infusion (BHI) broth (Qingdao Hepo Bio-Technology, China) supplemented with 5% fetal bovine serum (FBS; EXCELL BIO, China) until the optical density at 600 nm (OD₆₀₀) reached approximately 0.6. The MVs of *T. pyogenes* were extracted according to the manufacturer’s protocol for the MV extraction kit (RENGEN BIOSCIENCES, Liaoning, China). Briefly, the supernatant of fresh bacteria was collected by centrifugation at 5000 × *g* for 20 min at 4 °C. The supernatant was mixed with binding buffer, the binding resin was added, and the mixture was inverted to mix for 15 min. The pellet was harvested by centrifugation at 1500 × *g* for 2 min at RT. The supernatant was resuspended, transferred to a purification column, and then centrifuged at 2000 × *g* for 2 min at RT to discard the filtrate. After being washed twice, elution buffer was added to the column, which was subsequently centrifuged to collect the concentrated bacterial MV mixture. The purified MVs were stored at − 80 °C for further applications.

### Culture of bovine endometrial epithelial cells

BEECs were isolated from healthy uteri as previously described [19]. The BEECs were recovered and cultured in Dulbecco’s modified Eagle’s medium (DMEM)/F12 (Procell, Wuhan, China) supplemented with 10% FBS and maintained at 37 °C with 5% CO_2_. For treatment, BEECs were seeded into six-well plates at a density of 5 × 10^5^ cells/mL and cultured for 24 h before exposure to experimental treatments. The control group was left untreated, whereas the TP group was infected with *T. pyogenes* at a multiplicity of infection (MOI) of 100. After treatment for 6, 12, or 24 h, the cells were collected for subsequent experiments.

### Characterization of *T. pyogene*s-derived membrane vesicles

The physical properties of the isolated MVs were evaluated by dynamic light scattering (DLS) analysis with a NanoStar II system (Wyatt Technology, USA) to determine the average particle size and distribution. All measurements were performed in triplicate. The morphology of the MVs was further examined by scanning electron microscopy (SEM; JSM-IT700HR, JEOL, Japan). For SEM imaging, MV samples were fixed with 2.5% glutaraldehyde, postfixed with 1% osmium tetroxide, dehydrated through a graded ethanol series, and sputter-coated with gold before visualization.

### MV exposure experiments

To assess the effects of MVs on BEECs, the cells were seeded in six-well plates and allowed to adhere for 24 h. The cells were then treated with MVs at three different concentrations: 1 × 10^8^, 1 × 10^7^, and 1 × 10^6^ particles/mL. Each treatment was applied for 6, 12, or 24 h. Following treatment, the cells were harvested for subsequent analyses.

### ELISA

To determine cytokine production in cells treated with *T. pyogenes* and various concentrations of MVs, ELISA analysis was performed. The experimental samples were prepared by collecting the cell supernatant after treatment and centrifuging at 1000 × *g* for 10 min at 4 °C to remove cell debris. The supernatant was immediately used for IL-1β, IL-6, IL-18, and TNF-α ELISA (Jianglai, China) analysis. The absorbance at 450 nm was recorded with a microplate reader, and the cytokine concentrations were calculated on the basis of standard curves.

### Cell viability assay

BEECs were cultured in 96-well plates at a density of 2 × 10^4^ cells/mL. After treatment with different concentrations of MVs or *T. pyogenes*, cell viability was assessed using the Cell Counting Kit-8 (CCK-8; TransGen Biotech, China). Following incubation with CCK-8 reagent for 1–2 h at 37 °C, the absorbance at 450 nm was measured using a microplate reader. Each treatment was performed in triplicate.

### RNA isolation and real-time PCR

Total RNA was extracted from BEECs with TRIzol (Takara, Japan) according to the manufacturer's instructions. The purity and concentration of RNA were determined using a NanoDrop spectrophotometer (NanoDrop Technologies, Wilmington, DE, USA). Complementary DNA (cDNA) was synthesized using the Evo M-MLV Reverse Transcription Premixed Type Kit (AG, Hunan, China). Real-time PCR was performed using the ChamQ SYBR qPCR Master Mix Kit (Vazyme, Nanjing, China) on a CFX96 Real-Time PCR Detection System (Bio-Rad, USA). *GAPDH* was used as the internal reference gene. Relative gene expression was calculated using the 2^−ΔΔCt^ method. The primer sequences are listed in Table 1.

**Table 1:** The sequences of primers used for this study.

Gene	GenBank ID	Primer Sequence (5′-3′)	Product Length/bp
*GAPDH*	NM_001034034.1	F: GGACTTGAACTGTCTGCCCA R: CGACTTCAACAGCGACACTCAC	119
*GSDMD*	NM_001046160.3	F: AGCGTTCCAAAACAGACAACC R: CTCAGGATTCGAGGGGTGG	152
*IL-6*	NM_173923.2	F: CAGGAACGAAAGAGAGCTCCA R: GGGAGACAGCGAATGGAGTG	87
*IL-18*	NM_174091.2	F: ACCAGGGAAATCAACCTGTCT R: TGGTTACGGCCAGACCTCTA	110
*IL-1β*	NM_174093.1	F: GCCTACGCACATGTCTTCCA R: GTGCGTCACACAGAAACTCG	112
*MyD88*	NM_001014382.2	F: CTCATTGAGAAGAGGTGCCGT R: ATCAGTCGCTTCTGATGGGC	128
*NLRP3*	XM_010807177.4	F: CGTTAGTCTCTGCTTTTGCCTG R: TCCTGAGCCATGGAAGAAAGG	157
*NF-κB*	NM_001080242.2	F: GGGTGAATCGGAACTCTGGG R: GCCTGGTCCCGTGAAATACA	102
*TLR4*	NM_174198.6	F: GGGTGCGGAATGAACTGGTA R: TCCTGGATGATATTGGCGGC	115
*TNF-α*	XM_005223596.5	F: TCCAGAAGTTGCTTGTGCCT R: CACCTGGGGACTCTTCCCT	118

### Western blotting

Protein samples were lysed using RIPA buffer containing phenylmethanesulfonyl fluoride (PMSF; Solarbio, China). The total protein concentration was measured using a BCA assay kit. A 20 μg aliquot was resolved by SDS‒PAGE and transferred to PVDF membranes (Millipore, USA). The PVDF membranes were blocked with 5% BSA for 1 h at room temperature. The blots were incubated overnight at 4 ℃ with the following primary antibodies for detection: anti-NLRP3 (Proteintech, Wuhan, China, 1:1500), anti-ASC (Proteintech, Wuhan, China, 1:5000), anti-Caspase-1 (Proteintech, Wuhan, China, 1:2000), anti-GSDMD (Proteintech, Wuhan, China, 1:1000), anti-MyD88 (Proteintech, Wuhan, China, 1:5000), anti-P65 (Abcam, USA, 1:2000), anti-p-P65 (Abcam, USA, 1:1000), and anti-β-actin (Proteintech, Wuhan, China, 1:5000) antibodies. The membranes were subsequently incubated for 2 h at room temperature with either anti-mouse or anti-rabbit HRP-conjugated secondary antibodies (Proteintech, Wuhan, China; 1:5000). The protein bands were detected using a chemiluminescence detection system (Fujifilm, Japan) and quantified with ImageJ software (National Institutes of Health, Maryland, USA). The data were then normalized to β-actin as a loading control.

### Flow cytometry analysis

Apoptosis in BEECs was determined using an Annexin V-FITC/PI Apoptosis Detection Kit (TransGen Biotech, China) following the manufacturer’s protocol. In brief, after 24 h of treatment, BEECs were harvested and resuspended in annexin V binding buffer. Subsequently, 5 μL of Annexin V-FITC or PI was added separately, and the mixture was incubated for 15 min at room temperature in the dark. Afterwards, 200 μL of Annexin V binding buffer was added. No dye was added to the negative control. To obtain apoptosis-positive cells, half of the cells were treated in a 60 ℃ water bath for 10 min to induce apoptosis and mixed with the other half of the normal live cells. The mixed cells were divided into two groups, and 5 μL of Annexin V-FITC or PI was added for single staining. The samples were analysed using a flow cytometer (Becton, Dickinson and Company, USA).

### Scanning electron microscopy of BEECs

BEECs were seeded onto slides in 24-well plates. After 24 h, the cells were treated with MVs and *T. pyogenes* for 24 h. The cells were then fixed with 2.5% glutaraldehyde, washed and stored at 4 ℃. After being washed with PBS, the cells were collected and fixed with 1% osmium tetroxide at 4 °C for 12 h, followed by washing with distilled water and a graded ethanol series. The samples were then dried, sputter-coated with gold, and imaged using a scanning electron microscope (JSM-IT700HR, JEOL, Japan). SEM was used to investigate the impacts of *T. pyogenes* and MVs on BEECs.

### Statistical analysis of data

The data were analysed using GraphPad Prism 8.3 software and are expressed as mean ± SD. Student’s *t* test was used for two-group comparisons, whereas one-way ANOVA followed by Duncan’s multiple range test was used for multiple comparisons. *P* < 0.05 was considered significant, and *P* < 0.01 was considered highly significant. All the experiments were repeated at least three times.

## Results

### *T. pyogenes* induces inflammatory responses in BEECs

Cytokines are critical mediators of inflammation and serve as key indicators of immune activation. To investigate the inflammatory effect of *T. pyogenes* on BEECs, we measured both cytokine secretion and gene expression following stimulation.

**Figure 1:**
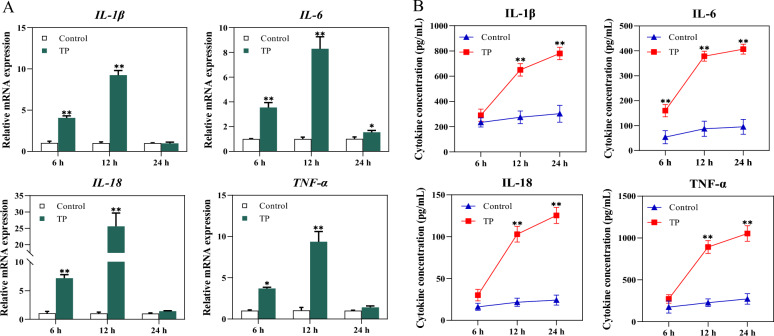
**Cytokine profile of BEECs after exposure to *T. pyogenes*.** **A** Relative mRNA levels of IL-1β, IL-6, IL-18, and TNF-α in BEECs collected 6, 12, and 24 h after *T. pyogenes* challenge were analysed via quantitative real-time PCR. **B** Cytokine concentrations in culture supernatants measured by ELISA. The data are presented as mean ± SD. *P < 0.05; **P < 0.01.

The results demonstrated that after 6 and 12 h of *T. pyogenes* treatment, the mRNA levels of IL-1β, IL-6, IL-18, and TNF-α were significantly increased (Figure 1A). These transcriptional changes largely mirrored the changes in secreted cytokine levels (Figure 1B), where the concentrations of IL-6, IL-18, and TNF-α markedly increased in the culture supernatant at 12 and 24 h (Figure 1B).

### Morphological and physical characterization of *T. pyogenes*-derived MVs

**Figure 2:**
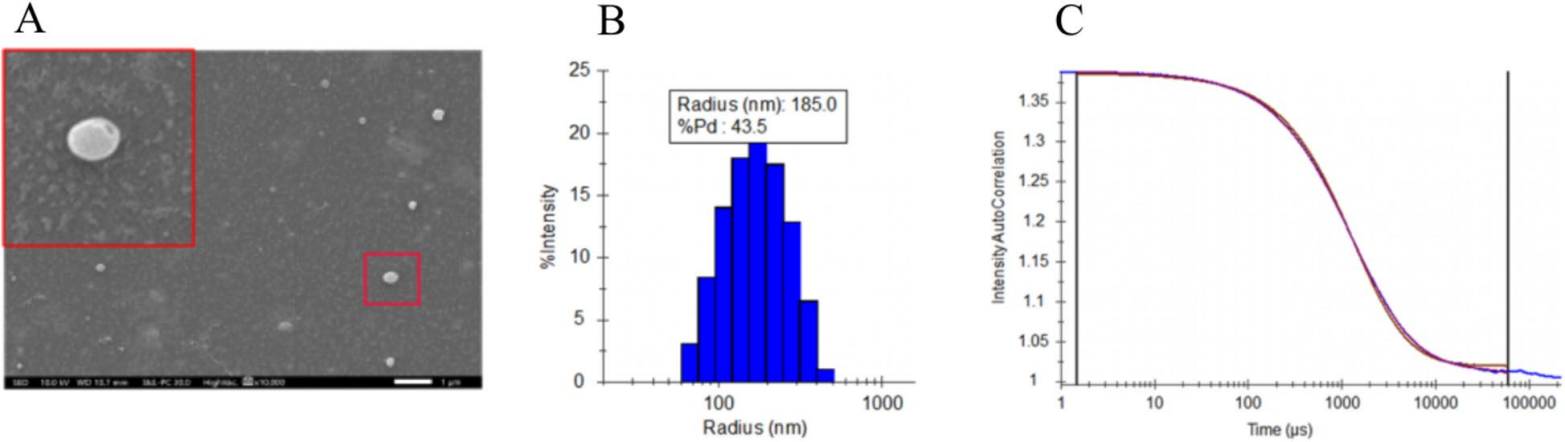
**Characterization of *T. pyogenes* membrane vesicles (MVs).** **A** SEM image showing the spherical morphology of MVs (10 000 × magnification). **B** Particle size distribution of MVs measured by dynamic light scattering (DLS). **C** DLS autocorrelation function confirming MV dispersion and stability.

To characterize the MVs from *T. pyogenes*, dynamic light scattering (DLS) and scanning electron microscopy (SEM) were employed. SEM imaging confirmed that the MVs had a spherical morphology with heterogeneous sizes ranging from 20 to 280 nm (Figure 2A). No residual bacterial structures, such as pili or cellular debris, were observed, indicating high purity of the vesicles. DLS intensity-weighted distribution revealed that the vesicles had a mean hydrodynamic radius of 185 ± 21 nm and a polydispersity index of 0.19. The polydispersity index (PDI) is the square of the polydispersity percentage (%PD); the more homogeneous the particle size is, the more stable the system. The PDI was in the range of 0.1–0.2, indicating a narrower particle size distribution and a more homogeneous system (Figure 2B). The intensity autocorrelation of MVs remains relatively high (1.4) at short lag times (less than 100 μs). After decay over a period of 100 000 μs, the curve approaches 1 at longer lag times (close to 100 000 μs), and the smoothness of the curve suggests that the particle size of MVs is uniform and stable in the solvent (Figure 2C). The average size and size distribution of the MVs strongly agreed with the SEM analysis results.

### MVs stimulate inflammatory cytokine production in BEECs

**Figure 3:**
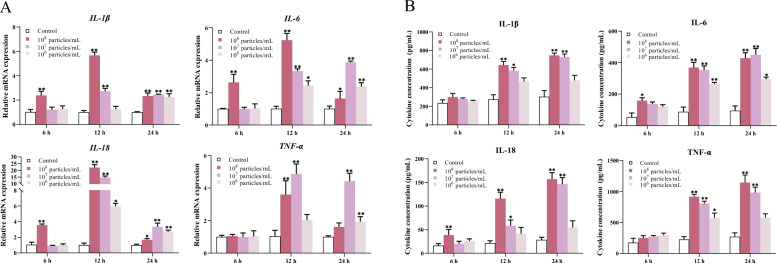
**Effects of MVs on cytokine gene and protein expression in BEECs after 6, 12, and 24 h of exposure.** **A** mRNA levels of IL-1β, IL-6, IL-18, and TNF-α were normalized to those of GAPDH. **B** ELISA quantification of cytokine levels in the supernatants. The data are presented as mean ± SD. *P < 0.05; **P < 0.01.

To explore the immunostimulatory potential of *T. pyogenes*-derived MVs, BEECs were treated with MVs at three concentrations: 1 × 10^8^, 1 × 10^7^, and 1 × 10^6^ particles/mL for 6, 12, and 24 h. The mRNA and protein levels of inflammatory cytokines were then assessed. As shown in Figure 3A, the expression of IL-1β, IL-6, IL-18, and TNF-α increased in a dose- and time-dependent manner. Notably, the MVs at a concentration of 1 × 10^8^ particles/mL consistently presented the highest levels of cytokine mRNA expression at all time points. For example, IL-6 and IL-18 were markedly upregulated as early as 6 h, with a sustained peak at 24 h in the high-dose group. Moderate concentrations (1 × 10^7^ particles/mL) induced moderate increases, whereas low concentrations (1 × 10^6^ particles/mL) elicited only mild responses, which were particularly evident at early time points. Similarly, cytokine protein secretion into the supernatant (Figure 3B) mirrored the transcriptional trends. The high-dose group (1 × 10^8^ particles/mL) presented the greatest increase in the protein levels of IL-6 and IL-18, followed by the moderate- and low-concentration MV groups. TNF-α and IL-1β secretion also increased in response to MV stimulation, with the high-dose group again showing the most robust effect. Interestingly, although high concentrations of MV consistently induced the strongest response, the differences between high and moderate concentrations narrowed over time for some cytokines (e.g., IL-18 and TNF-α at 24 h), suggesting that moderate MV exposure may be sufficient to elicit a sustained inflammatory effect without potential cytotoxicity associated with higher doses.

### Activation of the MyD88/NF-κB signalling pathway by MVs

**Figure 4:**
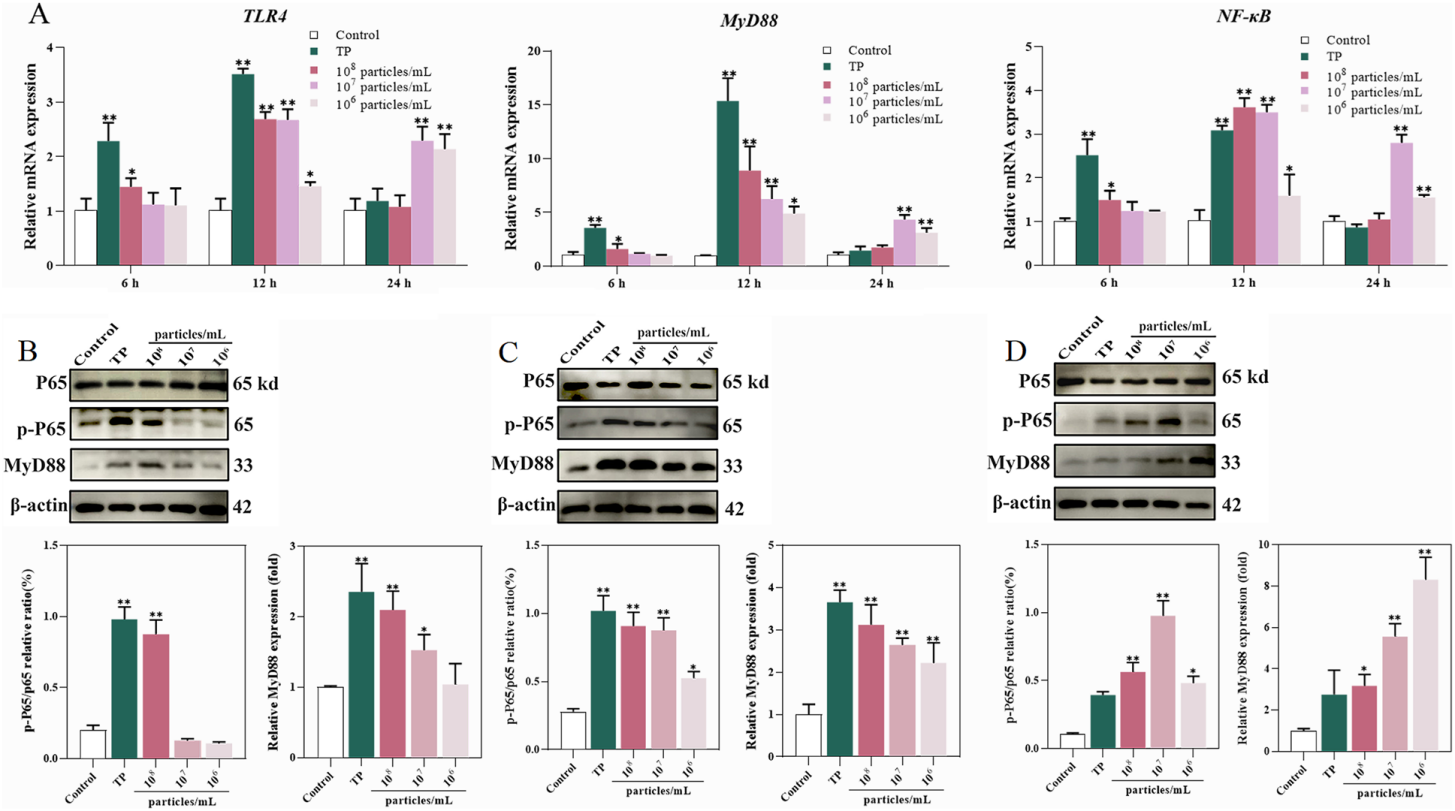
**Activation of the MyD88/NF-κB signalling pathway in BEECs challenged with* T. pyogenes* or MVs**. **A** mRNA levels of TLR4, MyD88, and NF-κB were assessed by qRT‒PCR. **B**–**D** Protein expression of MyD88 and phosphorylated P65 (p-P65) by western blot. The data are shown as mean ± SD. *P < 0.05; **P < 0.01.

BEECs were challenged with *T. pyogenes* and different concentrations of MVs for 6, 12, or 24 h. Then, the expression levels of TLR4, MyD88, and NF-κB at both the mRNA and protein levels were determined using qRT‒PCR and western blotting, respectively. Furthermore, the phosphorylation level of P65 was analysed to evaluate NF-κB activation. At 6 h posttreatment, the mRNA levels of TLR4, MyD88, and NF-κB were significantly elevated in the TP and high-dose (1 × 10^8^ particles/mL) groups (Figure 4A). By 12 h, all the treatment groups presented marked increases in these transcripts. Notably, at 24 h, only the moderate- (1 × 10^7^ particles/mL) and low-dose (1 × 10^7^ particles/mL) groups maintained significantly elevated mRNA expression of these key inflammatory markers, whereas the expression levels in the TP group decreased (Figure 4A). The results of the western blot analysis of MyD88 protein expression were consistent with the transcriptional trends (Figure 4B–D). With respect to NF-κB activation, phosphorylated P65 (p-P65) levels were significantly increased in the TP and high-dose groups (1 × 10^8^ particles/mL) after 6 h. Upon extended exposure for 12 h, all groups presented increased p-P65 protein expression. Interestingly, at 24 h, only the moderate- and low-dose groups maintained high p-P65 expression, whereas no significant change was observed in the TP group (Figure 4D). The dynamic pattern of P65 phosphorylation mirrored the expression of MyD88, suggesting that the MyD88/NF-κB signalling pathway is activated predominantly by MVs in a dose- and time-dependent manner.

### MVs promote inflammatory responses in BEECs via NLRP3 inflammasome activation

**Figure 5:**
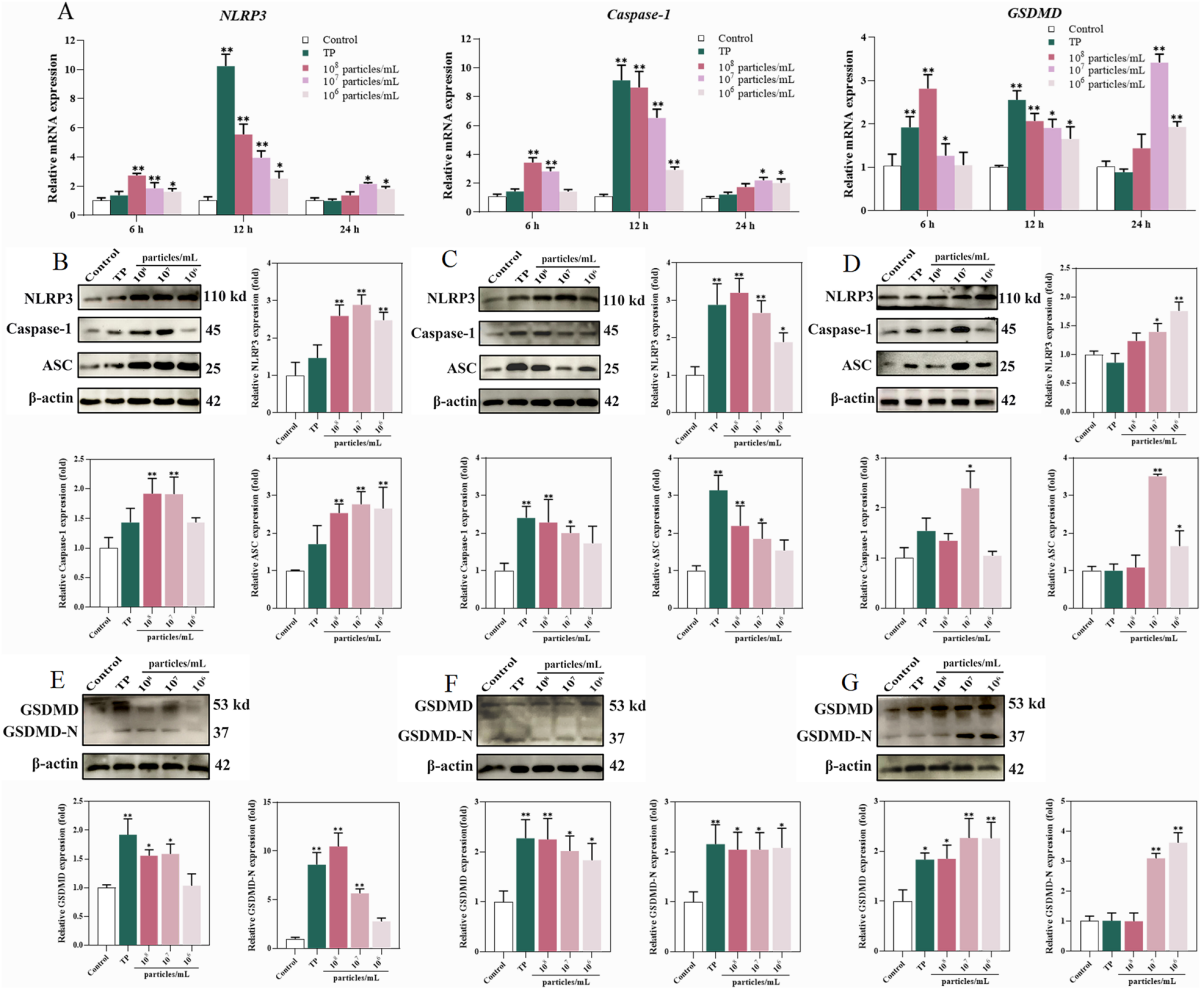
**Effects of *T. pyogenes* and MVs on inflammasome-related gene and protein expression in BEECs.** **A** mRNA expression of NLRP3, ASC, and caspase-1. **B**–**D** Western blot of the inflammasome components NLRP3, caspase-1, and ASC. **E**–**G** GSDMD and cleaved GSDMD-N protein levels. The data are shown as mean ± SD *P < 0.05; **P < 0.01.

Given the key role of the NLRP3 inflammasome in inflammatory endometrial disorders, we investigated the expression of NLRP3 and its downstream effectors, including ASC, Caspase-1, and GSDMD, following exposure to *T. pyogenes* and MVs. qRT‒PCR analyses revealed increased levels of NLRP3 mRNA in cells treated with different concentrations of MVs. qRT‒PCR analysis revealed that the mRNA expression of NLRP3, Caspase-1, and GSDMD was significantly increased in the TP and high-concentration MV (1 × 10^8^ particles/mL) groups at 6 h (Figure 5A). At 12 h, these genes remained elevated in the TP and high-concentration MV groups, and a moderate increase was also observed in the moderate-dose group. Interestingly, by 24 h, the expression levels in the TP group had decreased, whereas the level of the NLRP3 mRNA was significantly increased in the moderate- (1 × 10^7^ particles/mL) and low-dose (1 × 10^6^ particles/mL) groups (Figure 5A). The protein expression patterns were largely consistent with the transcriptional data. The western blot results revealed elevated levels of NLRP3, Caspase-1, and ASC in the TP and high-dose groups at 6 h and 12 h, whereas sustained or increased expression was detected in the moderate- and low-concentration groups at 24 h (Figure 5B–D). In addition, both GSDMD mRNA and total protein levels were significantly elevated in the TP and high-dose groups at 6 h and 12 h (Figure 5A, E, F). To assess GSDMD activation, the level of the cleaved form of GSDMD-N was also examined. A marked increase in GSDMD-N protein was observed in the TP and high-dose groups at 6 h and 12 h (Figure 5G), whereas at 24 h, GSDMD cleavage was prominent only in the moderate- and low-concentration groups (Figure 5G). These findings indicate that TP and high-dose MVs rapidly activate the NLRP3 inflammasome and induce pyroptosis, whereas medium and low concentrations of MVs elicit a delayed but sustained inflammasome response in BEECs. Notably, GSDMD cleavage did not fully correlate with Caspase-1 levels at later stages, suggesting that alternative pathways may contribute to GSDMD activation.

### Effects of* T. pyogenes* and MVs on the activity of BEECs

**Figure 6:**
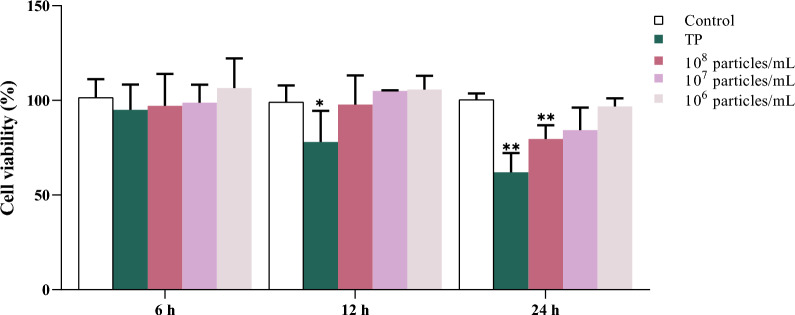
**BEEC viability after 6, 12, and 24 h of exposure to *T. pyogenes* or MVs, as assessed by a CCK-8 assay.** The data are presented as mean ± SD. *P < 0.05; **P < 0.01.

We found that the mRNA and protein expression of inflammatory pathway genes were inhibited by *T. pyogenes* and high concentrations of MVs at 24 h. To further investigate this phenomenon, a CCK-8 assay was employed. As shown in Figure 6, TP and high-concentration MVs (1 × 10^8^ particles/mL) significantly reduced cell viability in a time-dependent manner, with the most marked inhibition observed at 24 h. Conversely, moderate- (1 × 10^7^ particles/mL) and low-concentration (1 × 10^6^ particles/mL) MVs had no significant impact on cell viability compared with the control conditions.

### Distinct mechanisms of *T. pyogenes-* and MV-induced BEEC death

**Figure 7:**
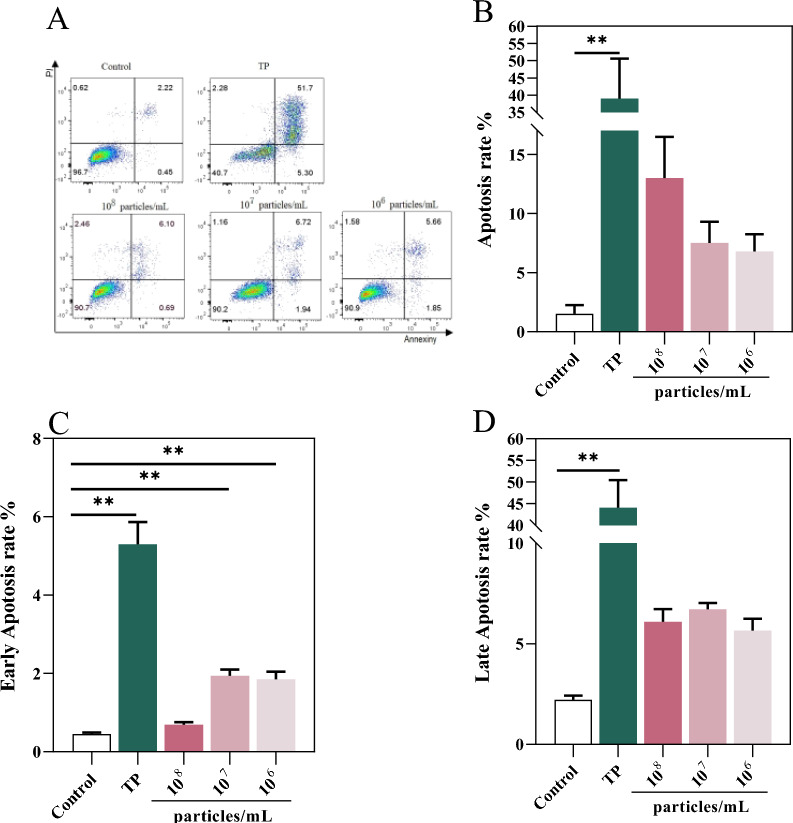
**Apoptosis of BEECs induced by *T. pyogenes* and MVs after 24 h.** **A** Apoptosis analysis by flow cytometry. **B**–**D** Quantification of total, early, and late apoptotic cells. The data are presented as mean ± SD. *P < 0.05; **P < 0.01.

We next used flow cytometry and SEM analysis to determine the type of cell death induced by *T. pyogenes* and MVs in BEECs. Flow cytometry based on Annexin V-FITC and PI staining revealed that at 24 h post-treatment, both early and late apoptosis rates were significantly greater in the TP group than in the control group (Figure 7A–D). Notably, the TP group exhibited a substantial proportion of late apoptotic or necrotic cells, indicating extensive membrane damage, while early apoptosis was also elevated. Similarly, MVs induced apoptosis to varying degrees depending on the concentration. Moderate- and low-concentration MV significantly elevated early apoptotic rates, whereas high-concentration MV caused a modest increase in both early and late apoptosis rates. However, the degree of apoptosis induced by MVs remained lower than that triggered by TP (Figure 7B–D).

**Figure 8:**
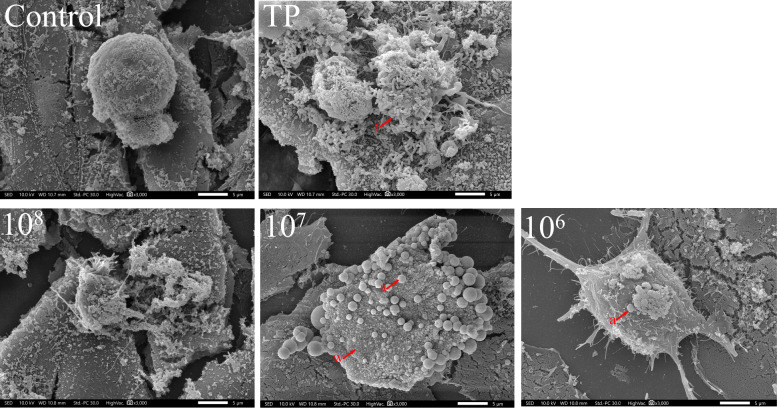
**SEM images of BEECs after 24 h of treatment with *T. pyogenes* or MVs** (3000 × magnification; scale bar = 5 μm). a: apoptotic body; t: *T. pyogenes*; w: cell membrane wrinkling.

SEM images further confirmed the distinct patterns of cell death induced by TP and MVs after 24 h of exposure (Figure 8). BEECs in the control group exhibited an intact morphology with smooth, rounded surfaces. In contrast, TP-treated cells presented severe membrane damage and extensive bacterial adhesion, indicating direct cytotoxic effects and potential lytic cell death. BEECs treated with high concentrations of MVs also presented disrupted membranes and collapsed cellular structures. Interestingly, cells treated with moderate and low concentrations of MVs maintained membrane integrity but displayed characteristic features of apoptosis, such as cell shrinkage, surface blebbing, and apoptotic body formation. These features were more prominent in the moderate-dose group than in the low-concentration MV group, which was consistent with the flow cytometry results. Collectively, these findings suggest that *T. pyogenes* induces BEEC death primarily through direct cytolysis and late apoptosis, whereas MVs—particularly at medium and low concentrations—predominantly promote early apoptosis with less pronounced necrosis or membrane damage.

## Discussion

This study aimed to investigate the mechanism of the inflammatory response and cellular damage to BEECs caused by *T. pyogenes* and its MVs. Our findings revealed that both *T. pyogenes* and its MVs activate the MyD88/NF-κB and NLRP3 inflammasome signalling pathways in BEECs. Notably, we demonstrated that *T. pyogenes* MVs trigger these pathways in a time- and dose-dependent manner, with early activation of NLRP3 at moderate to high MV concentrations and later activation of NF-κB and subsequent cytokine release at higher doses and longer exposure times.

Endometritis is a major postpartum disease in dairy cows that impairs milk yield and reproductive efficiency. *T. pyogene*s is a key pathogen in the development of endometritis [2, 20, 21]. Consistent with previous studies using endometrial organ cultures [22], our data revealed that BEECs exposed to *T. pyogenes* presented significantly elevated mRNA levels of the proinflammatory cytokines IL-1β, IL-6, IL-18, and TNF-α. Interestingly, MVs derived from *T. pyogenes* alone were also capable of modestly increasing both the mRNA and protein levels of these inflammatory cytokines in BEECs. Bacterial MVs have been implicated in numerous biological processes, including virulence, antibiotic resistance, intercellular communication, and immune modulation [23, 24]. However, *T. pyogenes*-derived MVs have not been characterized previously. In our study, scanning electron microscopy (SEM) revealed that the MVs were spherical with heterogeneous size distributions, whereas dynamic light scattering (DLS) revealed relatively uniform particle sizes ranging from 20–280 nm, which is consistent with reported features of bacterial MVs [25, 26]. Research has shown that bacterial MVs can induce mitochondria-mediated apoptosis and activate the NLRP3 inflammasome in immune cells [27]. Our data suggest that *T. pyogenes*-derived MVs may similarly contribute to the pathogenesis of endometritis by inducing inflammation in BEECs.

The MyD88/NF-κB and NLRP3 pathways are pivotal regulators of innate immune responses [28]. In our study, both *T. pyogenes* and its MVs activated these signalling cascades in BEECs. TLR4, a key pattern recognition receptor, recognizes pathogen-associated molecular patterns and activates downstream MyD88/NF-κB signalling [29]. After 6 h of *T. pyogenes* stimulation, BEECs presented significant upregulation of TLR4, MyD88, and NF-κB transcripts (*P* < 0.01). Similarly, high concentrations of MVs induced a comparable response (*P* < 0.05). NF-κB P65 is a critical subunit whose phosphorylation enhances the transcription of proinflammatory cytokines such as IL-6 and TNF-α [30]. Phosphorylated P65 was notably upregulated in the TP and high-concentration MV (1 × 10^8^ particles/mL) groups at 6 h, which correlated with elevated cytokine levels in the supernatant. This finding is consistent with a previous study reporting the cytotoxic effects of *T. pyogenes* on porcine bronchial epithelial cells after prolonged exposure [31]. We observed significant cell death in BEECs after 24 h of *T. pyogenes* stimulation, potentially explaining the decline in inflammatory gene expression at that time point.

We also demonstrated that the NLRP3 inflammasome was activated by both *T. pyogenes* and its MVs, although the latter triggered activation more rapidly. NLRP3 is a cytosolic sensor that forms a multiprotein inflammasome complex in response to microbial and damage-associated signals [32, 33]. After 6 h, all MV-treated groups presented marked upregulation of NLRP3 at both the mRNA and protein levels, whereas such changes in the TP group were not observed until 12 h, mirroring the activation patterns of caspase-1 and ASC. Activation of the inflammasome requires a sensor (e.g., NLRP3), an adaptor protein (ASC), and an effector caspase (typically caspase-1) [32]. MVs may enter cells more readily because of their lipid composition, possibly accounting for their more rapid activation of NLRP3, a hypothesis that warrants further study. Inflammasome assembly leads to caspase-1 activation, which cleaves pro-IL-1β and pro-IL-18 into their mature forms [34] and processes gasdermin D (GSDMD), whose N-terminal fragment (GSDMD-N) forms membrane pores that facilitate cytokine release [35, 36]. Notably, GSDMD expression and cleavage were significantly increased in the TP group at 6 h, suggesting early pyroptotic activity, possibly mediated by other caspase family members. At 24 h, elevated GSDMD and GSDMD-N levels were observed only in the moderate- (1 × 10^7^ particles/mL) and low-dose (1 × 10^6^ particles/mL) groups, which was consistent with sustained inflammasome activation and cytokine release. These data collectively support the conclusion that both the TLR4/MyD88/NF-κB pathway and the NLRP3 inflammasome play critical roles in mediating the inflammatory response of BEECs to *T. pyogenes* and its MVs, corroborating earlier findings [22, 29]. In summary, *T. pyogenes* and its MVs exhibit time- and dose-dependent activation of the TLR4/MyD88/NF-κB and NLRP3 inflammasome signalling pathways. *T. pyogenes* and 1 × 10^8^ particles/mL of MVs can activate the TLR4/MyD88/NF-κB pathway earlier, whereas the former activates the NLRP3 inflammasome relatively late. In contrast, 1 × 10^6^ particles/mL MVs activated NLRP3 inflammatory vesicles earlier. Previous studies have shown that attenuating the activation of the NLRP3 inflammasome can protect against pyroptosis and bovine endometritis [37]. Therefore, the inhibition of *T. pyogenes* vesicle secretion as well as the blockade of MyD88/NF-κB and NLRP3 pathway activation provide novel therapeutic targets for bovine endometritis and other inflammatory diseases.

Furthermore, our study revealed differential cell damage patterns depending on the stimulus. After 24 h of incubation, *T. pyogenes* and high concentrations of MVs (1 × 10^8^ particles/mL) caused marked reductions in BEEC viability (*P* < 0.01), accompanied by pronounced necrosis. In contrast, the moderate-dose (1 × 10^7^ particles/mL) group presented a significant increase in the number of apoptotic cells, as shown by flow cytometry (*P* < 0.01), while SEM imaging revealed the presence of apoptotic bodies. Notably, although SEM micrographs revealed evident membrane disruption in BEECs exposed to high concentrations of MVs, flow cytometric analysis did not reveal a notable increase in the number of apoptotic cells. These findings suggest that while high concentrations of MVs induce other modes of cell death, such as pyroptosis or necrosis, moderate concentrations may preferentially induce apoptosis. This is particularly noteworthy because previous studies have suggested that *T. pyogenes* does not induce apoptosis [38], highlighting a novel aspect of MV-mediated cytotoxicity that merits further investigation.

Despite extensive cell death in the TP and high-dose groups at 24 h, the cytokine concentrations in the culture supernatant remained elevated (*P* < 0.01). This could be attributed to cumulative cytokine release prior to cell death, a phenomenon observed in macrophages exposed to LPS [39]. These findings indicate that while *T. pyogenes* and its MVs both provoke inflammatory responses, MVs exhibit dose-dependent effects and may preferentially induce apoptosis at moderate concentrations.

This increase is attributable to the occurrence of inflammatory responses in all groups at these time points, leading to the accumulation of cytokines in the supernatant. Consequently, even though the TP and high-concentration MV (1 × 10^8^ particles/mL) groups did not exhibit an overt inflammatory response at 24 h, the cytokine expression in the supernatant still tended to increase. Considering the mechanisms of inflammation in BEECs discussed earlier together, it can be hypothesized that both *T. pyogenes* and its MVs can trigger inflammatory responses in BEECs, with specific concentrations of MVs potentially mediating apoptosis.

In conclusion, our findings suggest that *T. pyogenes* and its MVs activate inflammatory responses in BEECs via the TLR4/MyD88/NF-κB and NLRP3 inflammasome pathways. Compared with whole bacteria, MVs may facilitate earlier and more sustained activation of these pathways, with distinct effects on cell fate depending on their concentration. These findings contribute to a deeper understanding of the pathogenic mechanisms underlying *T. pyogenes*-induced endometritis and highlight bacterial MVs as potential targets for future therapeutic interventions.

## Data Availability

The data that support the findings of this study are available in the Materials and Methods, Results section of this article.
